# Voxel-based morphometry reveals immune-metabolic dysregulation driving adaptive cortical gray matter remodeling in patients with esophageal cancer

**DOI:** 10.3389/fonc.2025.1625625

**Published:** 2025-11-13

**Authors:** Kaihua Zhang, Wei Su, Jiayu Lin, Yingli Gao, Chunming Gu, Ye Li, Ning Jiang, Yingtao Hao, Hai Zhong, Weiquan Zhang

**Affiliations:** 1 Faculty of Psychology, Shandong Normal University, Jinan, China; 2 Shandong Provincial Key Laboratory of Brain Science and Mental Health, Shandong Normal University, Jinan, China; 3 Department of Radiology, Mayo Clinic, Rochester, MN, United States; 4 Department of Pediatrics, The Second Hospital, Cheeloo College of Medicine, Shandong University, Jinan, China; 5 Department of Thoracic Surgery, The Second Hospital, Cheeloo College of Medicine, Shandong University, Jinan, China; 6 Department of Radiology, The Second Hospital, Cheeloo College of Medicine, Shandong University, Jinan, China

**Keywords:** esophageal cancer, voxel-based morphometry, immune-metabolic dysregulation, gray matter, structural magnetic resonance imaging

## Abstract

Esophageal cancer (EC), a highly prevalent malignant cancer, is frequently accompanied by cancer-related cognitive impairment (CRCI), yet its underlying neural mechanisms remain poorly understood. This study integrated inflammatory biomarkers and nutritional index with structural magnetic resonance imaging (MRI) to investigate the characteristics of brain structural alterations in EC patients and their association with systemic inflammation and nutritional metabolism. A total of 49 treatment-naive EC patients and 31 healthy controls (HC) were enrolled. High-resolution T1-weighted MRI scans and peripheral blood indices (including platelet-to-lymphocyte ratio (PLR), neutrophil-to-lymphocyte ratio (NLR)) were collected. Voxel-based morphometry (VBM) was employed to assess gray matter (GM) volume differences, and correlations between GM volume, inflammatory markers and nutritional index were analyzed. Results demonstrated that the EC group exhibited significantly elevated monocyte counts and PLR, alongside reduced lymphocytes, albumin levels, and prognostic nutritional index compared to HC (*p* < 0.05). Structural MRI revealed significantly increased GM volume in bilateral occipital lobes, basal ganglia, pre-/postcentral gyri, and the right temporal lobe in EC patients, and decreased GM volume in bilateral parahippocampa gyri, amygdala, and cerebellum Posterior Lobe (FDR correction, *p* < 0.05). Partial correlation analysis indicated a negative association between GM volume in the right basal ganglia and PLR (r = - 0.464, *p* = 0.005). These findings suggest that brain structural alterations in EC patients may be driven by systemic inflammation and nutritional imbalance, reflecting a dynamic equilibrium between neuroplastic compensation and neuroinflammatory injury. The negative correlation between GM volume and PLR provides neuroimaging evidence for inflammation-mediated CRCI mechanisms, offering novel targets for the development of early intervention strategies.

## 1 Introduction

Esophageal Cancer (EC) is a prevalent malignant neoplasm worldwide, with the seventh highest global incidence and sixth highest mortality (1). In China, EC poses an exceptionally high disease burden, accounting for more than half of the global cases, and 90% of which are Esophageal Squamous Cell Carcinoma (ESCC) (2). The majority of patients are diagnosed at middle and advanced stages, contributing to a mortality rate in China that is twice the global average, thereby presenting substantial challenges for disease management and prevention (3). The malignant development of EC is a complex process involving multifactorial, multistage and multi-signal pathway regulation (4, 5). It is worth noting that cancer patients often suffer from cognitive dysfunction, known as cancer-related cognitive impairment (CRCI). CRCI not only severely diminishes the quality of life of patients, but also causes great damage to their daily function, self-identity and professional ability (6). As cancer treatment technologies advance and patient survival rates increase, in-depth analysis of the neurobiological mechanism of CRCI has become an urgent need to improve the cognitive prognosis of patients.

Studies have shown that the development of EC is closely related to inflammation, which promotes the development of EC through various mechanisms, including oxidative damage, epigenetic changes, and immunosuppression (7, 8). In recent years, the role of inflammatory mechanisms in neurodegenerative diseases and cognitive impairment has attracted increasing attention (9, 10). Research indicates that chronic inflammation and abnormal immune response can cause oxidative stress and increase of proinflammatory cytokines in the body, interfere with synaptic plasticity and neurogenesis, and eventually lead to brain structural atrophy and cognitive impairment (11). Inflammatory markers, such as Neutrophil-to-Lymphocyte Ratio (NLR) and Platelet-to-Lymphocyte Ratio (PLR), serve as sensitive indicators of systemic inflammation. Their elevation partially reflects the aggravation of oxidative stress and inflammatory response. Previous studies have shown that NLR and PLR are associated with the severity and prognosis of a variety of cognitive-related diseases, and may serve as predictors of cognitive impairment (12, 13). Marsland et al. demonstrated that inflammation was associated with the weakening of short-term memory, language ability, spatial reasoning ability and executive ability, as well as reductions of cerebral cortex, hippocampal volume and cortical surface area (14). Peripheral inflammation may enter the central nervous system (CNS) through various ways, triggering neuroinflammatory cascades. The content of proinflammatory cytokines and their receptors in the hippocampus and cerebral cortex is relatively high, which makes the hippocampus more vulnerable to inflammatory attack. This interferes with neurogenesis, disrupts neurotransmission, damages synaptic plasticity and causes dendritic branch atrophy, ultimately leading to neuronal damage, brain network remodeling and cognitive dysfunction (15). However, neuroimaging studies on CRCI in EC patients remain limited, and it is still unclear whether inflammation mediated brain structural changes are involved in the cognitive decline.

Structural Magnetic Resonance Imaging (MRI) technology provides an important tool for noninvasive analysis of brain structural abnormalities. Because of the unique cancer biological characteristics (such as high invasiveness and easy metastasis) and treatment modes (such as concurrent chemoradiotherapy), the brain structural changes of EC patients may present a specific pattern. Based on this, this study intends to systematically explore the characteristics of structural changes in brain regions of EC patients using high-resolution MRI technology. By incorporating dynamic monitoring of inflammatory markers (such as NLR, PLR), we seek to reveal the intrinsic association between inflammation and brain structure, thereby providing a novel perspective for early warning and targeted intervention of CRCI.

## 2 Materials and methods

### 2.1 Subjects

There are two groups of data reported in this study: 49 initially diagnosed EC patients with eating obstruction for 2 to 3 months (Mean age = 64.63 years, range = 49-80 years, 8 females) and 34 healthy controls (HC) (Mean age = 61.97 years, range = 50-82 years, 15 females). All subjects were recruited between July 2020 and September 2024. Two radiologists independently assessed and determined that all EC patients were at stage IIB and IIIB. Participants were excluded from the final analysis if they met any of the following criteria: (1) contraindications to MRI scanning (e.g., presence of metallic implants or severe claustrophobia); (2) clinically significant abnormalities detected during neurological or physical examinations; (3) a history of traumatic brain injury; or (4) any current or past diagnosis of psychiatric or neurological disorders. This study has been approved by the ethics committee, and all subjects provided informed consent before enrollment.

### 2.2 MRI acquisition

All data were acquired in a GE MRI Discovery 750w 3.0 T (GE, USA) at the Second Hospital of Shandong University, Jinan, China. The instrument is equipped with a 64-channel head coil. All subjects were placed in a supine position and used custom foam pads to reduce head movement. High-resolution 3D-T1 weighted structural images were collected using a gradient echo pulse sequence. Acquisition parameters are as follows: Echo Time (TE) = 2.34 ms, Repetition Time (TR) = 2530 ms, Inversion Time (TI) = 1100 ms, 256 × 256 matrix, field of view = 256 × 256 mm^2^, flip angle = 7°, number of slices = 192, slice thickness = 1.0 mm, total sequence duration = 363 s.

### 2.3 Clinical data analysis

We collected clinical data from all subjects, including height, weight, education, lifestyle factors (smoking, alcohol consumption) and clinical indicators such as differential blood cell count and albumin levels. SPSS 24 was used to perform independent-samples *t* test on clinical data. A two-tailed *p*-value < 0.05 was considered statistically significant.

### 2.4 Data preprocessing

Data were analyzed using SPM12 (http://www.fl.ion.ucl.ac.uk/spm/software/spm12) based on MATLAB 2015a (MathWorks, Natick, Ma, USA) extension toolbox CAT12 (16). Before preprocessing, the structural MRI images were evaluated for artifacts by SPM12, and the central point was repositioned on the anterior commissure. The default settings were used in this study based on the CAT12 Toolbox Manual (http://www.neuro.uni-jena.de/cat12/CAT12- Manual.pdf). Following initial quality assurance procedures, structural images underwent intensity inhomogeneity correction and were subsequently partitioned into cerebral tissue compartments (gray matter (GM), white matter, and cerebrospinal fluid) through probabilistic classification. Spatial alignment to the standardized MNI coordinate system was achieved through a multi-stage registration process, which included both affine transformations and diffeomorphic field estimation within an integrated computational framework incorporating high-dimensional diffeomorphic anatomical registration through exponentiated lie algebra (17). Tissue probability maps were then intensity-modulated through application of Jacobian determinant scaling factors derived from the deformation fields to preserve quantitative volumetric information. The adjusted GM density maps underwent spatial filtering using an isotropic Gaussian smoothing kernel with 8-mm full-width at half-maximum (FWMH) to facilitate subsequent group-level analysis. Quantitative volumetric measurements of cerebral tissues were computed through numerical integration of the binarized segmentation outputs. Total intracranial volume (TIV) was calculated as the composite sum of GM, white matter, and cerebrospinal fluid volumes, and all GM volume measurements were subsequently normalized to TIV. These procedures were implemented automatically within the CAT12 pipeline. In addition, although the CAT12 processing suite incorporates advanced denoising methodologies, all volumetric images underwent systematic visual verification to mitigate residual artifact interference. Following the exclusion of ineligible subjects, the resulting volumetric images from the remaining 34 healthy controls and 41 EC patients exhibited satisfactory homogeneity.

### 2.5 Statistical analysis

To investigate intergroup differences, age, gender, education, lifestyle factors (smoking, alcohol consumption) and Body Mass Index (BMI) were taken as covariates and two-sample *t*-test was conducted. An absolute threshold mask (excluding all voxels with GM values less than 0.01) was used to avoid edge effects. The EC-HC results were FDR corrected at the voxel level with a cluster size > 20 voxels, and a threshold of *p* < 0.05 was reported.

To test whether the changes in GM volume between groups were related to clinical data, we performed an exploratory correlation analysis using Spearman’s correlation coefficient implemented in SPSS 24. First, the brain regions with significant differences between groups were defined as regions of interest (ROI) using xjview (http://www.alivelearn.net/xjview). Then, the average GM changes of all subjects in ROIs were extracted by Marsbar. SPSS 24 was used to analyze the partial correlation between GM changes and clinical data, with age, gender, education, lifestyle factors (smoking, alcohol consumption) and BMI included as covariates. Considering the relatively small sample size and exploratory nature of the analysis, no multiple comparative correction was performed. The results were reported at a threshold of *p* < 0.01.

## 3 Results

### 3.1 Demographic and clinical characteristics of patients with EC and HC

Clinical data between the two groups were compared using independent-samples *t*-test. We found that EC group showed significant differences (*p*-values < 0.05) from HC group in gender, BMI, monocytes, lymphocytes, albumin, PLR, lymphocyte-monocyte ratio (LMR), systemic immunity and prognostic nutrition (see **Table 1**).

**Table 1:** Comparison of clinical data between EC group and HC group.

Dimensions	EC	HC	T value	*P*-value (two-tailed)
Number of people	41	34	–	–
Gender (males/females)	33/8	19/15	5.29	0.021
Age	64.63 ± 6.95	61.97 ± 7.00	1.61	0.113
Body mass index (BMI)	23.04 ± 3.17	24.90 ± 2.67	-2.72	0.008
Weight (kg)	62.44 ± 9.30	68.53 ± 11.87	-2.39	0.020
Height (cm)	164.61 ± 6.72	165.74 ± 8.85	-0.60	0.548
Smoking (Yes/No)	15/26	11/23	0.15	0.701
Alcohol consumption (Yes/No)	18/23	14/20	0.06	0.812
Education	8.41 ± 2.92	8.79 ± 1.47	-0.73	0.470
Platelet	247.81 ± 78.55	214.61 ± 60.15	1.96	0.054
Monocytes	0.56 ± 0.22	0.43 ± 0.13	2.98	0.004
Lymphocyte	1.54 ± 0.52	2.30 ± 1.76	-2.63	0.010
Neutrophi	4.40 ± 1.60	4.81 ± 9.37	-0.27	0.786
Albumin	40.97 ± 3.92	44.65 ± 3.12	-4.29	<0.001
Neutrophils-lymphocyte ratio (NLR)	3.21 ± 2.14	2.42 ± 4.50	0.99	0.325
Platelet-lymphocyte ratio (PLR)	174.69 ± 65.43	117.66 ± 56.62	3.88	<0.001
Lymphocyte-monocyte ratio (LMR)	3.04 ± 1.20	5.29 ± 2.16	-5.24	<0.001
Systemic immunity-inflammatory index (SII)	784.05 ± 510.82	457.52 ± 593.21	2.51	0.015
Prognostic nutritional index (PNI)	48.65 ± 4.56	56.14 ± 8.51	-4.81	<0.001
Geriatric nutrition risk index (GNRI)	104.67 ± 8.76	102.66 ± 34.78	0.32	0.755

EC, esophageal cancer; HC, healthy controls.

### 3.2 VBM analysis in patients with EC and HC

**Figure 1:**
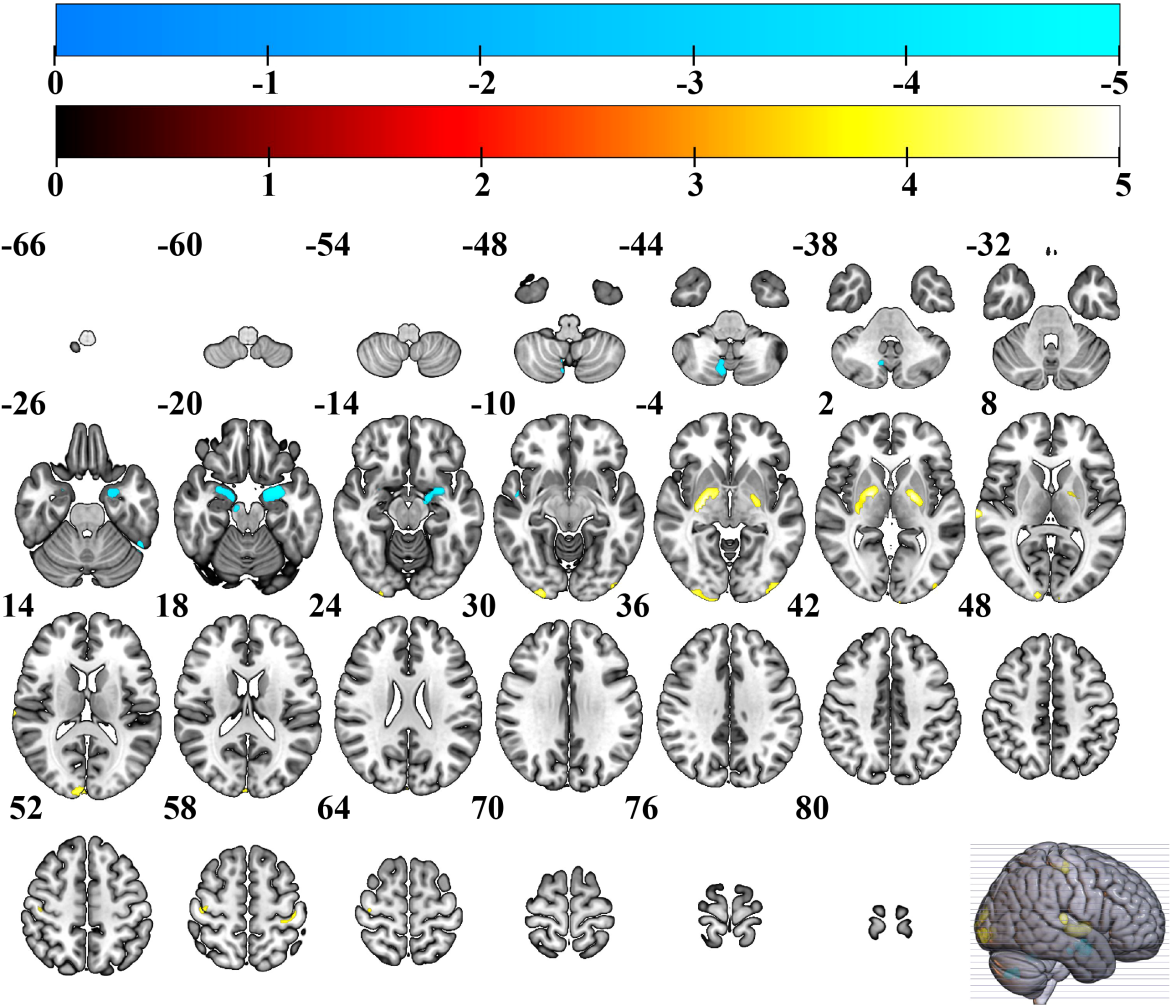
Brain regions with increased GM volume in EC group. Warmer colors show brain regions with increased GM volume in EC group compared to HC group. EC, esophageal cancer; HC, healthy controls; GM, gray matter.

The results of the two-sample *t*-test showed that the GM volume in EC group was significantly higher than HC group in bilateral occipital gyrus (extending to the middle occipital gyrus and inferior occipital gyrus), bilateral basal ganglia (extending to the lentiform nucleus, putamen, medial and lateral globus pallidus), bilateral precentral gyri, postcentral gyrus. GM volume also increased significantly in the right lingual gyrus, and right superior temporal gyrus. However, the EC patient group also exhibited regions of reduced GM volume, predominantly involving the left and right parahippocampal gyri, left and right amygdala, left and right superior temporal gyri, left and right cerebellum posterior lobe, as well as the right midbrain (see **Table 2**, **Figure 1**).

**Table 2:** Brain regions with significant differences in GM volume between EC group and HC group.

Clusters	Regions	BA	Voxels	X	Y	Z	*T*	*P_FDR-corr_*	*Effect Size*
EC-HC									
1	Right Lentiform Nucleus Right Putamen Right Medial and Lateral Globus Pallidus	-	761	16	-6	0	5.39	0.026	1.32
2	Left Lentiform Nucleus Left Putamen Left Medial and Lateral Globus Pallidus	-	444	-22	-8	0	4.93	0.026	1.20
3	Right Superior Temporal Gyrus	42	180	69	-22	9	4.90	0.026	1.20
4	Right Cuneus	17/18	28	4	-104	-4	4.61	0.026	1.13
5	Right Cuneus Right Middle Occipital Gyrus	-	256	14	-99	10	4.57	0.026	1.12
6	Right Precentral Gyrus Right Postcentral Gyrus	3/4	120	45	-21	56	4.50	0.027	1.10
7	Right Lingual Gyrus	18	32	3	-98	-21	4.36	0.030	1.07
8	Right Middle Occipital Gyrus Right Cuneus Right Inferior Occipital Gyrus Right Lingual Gyrus	18/19	554	27	-98	-9	4.36	0.030	1.07
9	Left Middle Occipital Gyrus Left Inferior Occipital Gyrus	18/19	247	-45	-92	-6	4.24	0.032	1.04
10	Left Postcentral Gyrus Left Precentral Gyrus	3/4	85	-45	-26	57	4.06	0.035	0.99
11	Left Cuneus	18	24	-8	-105	4	3.86	0.040	0.94
HC-EC									
1	Left Parahippocampa Gyrus Left Amygdala Left Superior Temporal Gyrus	34/28	837	-28	0	-20	5.85	0.004	1.43
2	Left Cerebellum Posterior Lobe	37	121	-51	-54	-27	5.15	0.005	1.26
3	Right Parahippocampa Gyrus Right Amygdala	34/28	297	27	2	-20	4.53	0.016	1.11
4	Right Cerebellum Posterior Lobe	-	264	6	-75	-42	4.47	0.017	1.09
5	Right Midbrain	-	49	10	-16	-21	4.31	0.024	1.05
6	Right Superior Temporal Gyrus	38	22	50	-4	-10	4.18	0.029	1.02

GM, gray matter; EC, esophageal cancer; HC, healthy controls; BA, Brodmann area. X, Y, Z = MNI coordinates. The threshold was set at *p* < 0.001 uncorrected at the cluster wise level with a cluster size > 20 voxels and *p* < 0.05 with FDR correction at the voxel level.

### 3.3 Associations between clinical assessments and structural parameters in EC patients

**Figure 2:**
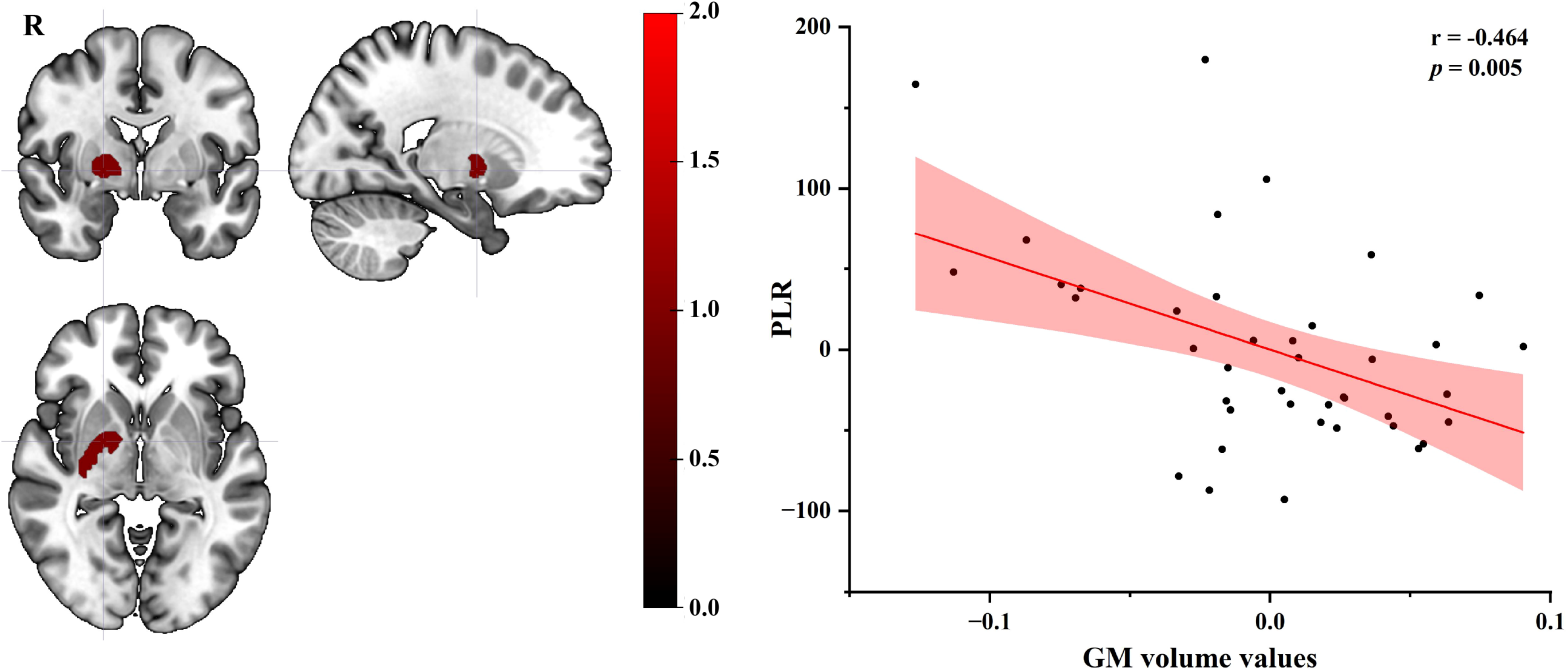
Partial correlation plot of GM volume values in the right basal ganglia of EC group with PLR score (age, gender, education, lifestyle factors and BMI as covariates). GM, gray matter; EC, esophageal cancer; PLR, platelet-lymphocyte ratio; BMI, Body Mass Index; R, right.

Using age, gender, education, lifestyle factors (smoking, alcohol consumption) and BMI as covariates, partial correlation analysis showed that GM volume values of the right basal ganglia (extending to the lentiform nucleus, putamen, medial and lateral globus pallidus) in EC patients were negatively correlated with PLR scores (r = - 0.464, *p* = 0.005) (see **Figure 2**).

## 4 Discussion

This study, for the first time, reveals the unique pattern and underlying mechanism of brain structural changes in EC patients, by integrating inflammatory-immune and nutritional metabolic indicators and brain imaging data. Compared with HC group, EC group exhibited systemic immune imbalance (monocytosis, lymphopenia, PLR/LMR abnormalities) and nutritional metabolism disorder (PNI reduction), accompanied by significant increases in GM volume in broad brain regions such as bilateral occipital gyrus, basal ganglia, middle frontal gyrus. The GM volume in the right basal ganglia was negatively correlated with PLR score in EC patients. These findings suggest that CRCI in EC patients may not be a simple neuronal degenerative change but a complex dynamic process involving inflammation-mediated neuroplasticity and adaptive compensation.

This study is also the first to uncover the complex interaction between immune-nutritional imbalance and the structural remodeling of CNS in EC patients. The patients showed mononucleosis, lymphopenia and PLR/LMR abnormalities. The human blood system contains a variety of inflammatory cells, such as neutrophils, lymphocytes, monocytes and platelets, which are effective prognostic factors for patients with malignant tumors (18–21). Therefore, the findings of mononucleosis, lymphopenia and PLR/LMR abnormalities may indicate the formation of systemic inflammation and immunosuppressive microenvironment. The role of systemic inflammatory response in tumors is not clear. Systemic inflammatory response can either promote or inhibit the occurrence and progression of tumors, and even influence the responsiveness of patients to systemic anti-tumor therapy (22). Furthermore, the tumor microenvironment increases the likelihood of tumor metastasis, thereby accelerating the progression of the disease (23).

The changes of systemic inflammation and immunosuppressive microenvironment in EC patients may drive CNS changes through multiple pathways. Firstly, elevated monocytes may differentiate into pro-inflammatory tumor-associated macrophages, release cytokines such as IL-6, activate microglia through blood-brain barrier leakage or vagus nerve signal transmission, and trigger astrogliosis and local edema (such as increased GM volume in basal ganglia) (24–27). Secondly, a significant decrease in lymphocytes (1.54 ± 0.52 vs 2.30 ± 1.76, *p* = 0.010) may impair the anti-tumor immune response while reducing neurotrophic factor secretion (28). Therefore, immune cells may regulate neuroplasticity through secreting cytokines (29). In addition, PNI reflects the nutritional and immune status by calculating the albumin level and total lymphocyte count in peripheral blood. The nutritional status of cancer patients is closely related to patients’ long-term survival (30, 31), and its significant reduction (48.65 vs 56.14, *p* < 0.001) suggests that EC patients have nutrition and immunity dysfunction. Albumin is the key carrier protein for the synthesis of neurotrophic factors. Its deficiency may affect the transport and stability of brain-derived neurotrophic factors, and then damage the survival of neurons, synaptic plasticity, and functional compensation. These findings highlight the central role of the “inflammation-metabolism-nerve” axis in esophageal CRCI, providing a new supplement to the traditional degenerative theory.

In this study, significant increases in GM volume were observed in multiple brain regions in EC patients, including bilateral basal ganglia, anterior and posterior central gyrus, inferior middle temporal gyrus, and inferior middle occipital gyrus. The distribution pattern suggests a complex interaction between systemic pathophysiological processes and local adaptive compensation. Bilateral basal ganglia (lenticular nucleus, putamen, globus pallidus) serve as the core hub for motor control and cognitive flexibility (32, 33), and their volume increase may be related to various factors. EC patients were accompanied by increased levels of proinflammatory factors, which may trigger central nervous inflammation through blood-brain barrier disruption (34), activate microglia (35), and lead to the remodeling of basal ganglia structure (36). As key regions of primary motor and sensory cortex, the anterior and posterior central gyrus regulate swallowing, limb movement and somatosensory function (37, 38). EC patients often suffer from motor sensory dysfunction due to dysphagia, pain or side effects from treatment (such as chemotherapy-induced peripheral neuropathy) (39), which may trigger compensatory remodeling of the CNS. There is increasing evidence that many chronic pain-related diseases exhibit extensive brain function and structural reorganization, such as primary sensory cortex, primary motor cortex, anterior cingulate gyrus and lenticular nucleus (40–42). For example, long-term dysphagia may increase the demand for muscle control in the throat by the anterior central gyrus (motor area), and promote GM volume increase through synapse regeneration or glial cell proliferation to maintain functional compensation (43, 44). As the primary auditory cortex, the GM volume of the middle and inferior temporal gyrus in EC was significantly increased compared with that in HC, potentially reflecting complex neural remodeling mechanisms in the course of the cancer. The GM volume of the middle and inferior occipital gyrus in EC group was significantly increased compared with that in HC, which may indicate that specific adaptive or pathological remodeling occurred in the CNS during cancer progression. The middle and inferior occipital gyrus are the core brain region of visual information processing (45). And the GM structural changes of their primary visual cortex and visual association cortex may be related to the compensation mechanism of visual perception induced by long-term nutritional metabolism disorders or chronic pain in EC patients.

However, the EC patient group also exhibited regions of reduced GM volume, predominantly involving the left and right parahippocampal gyri, amygdala, superior temporal gyri, cerebellum posterior lobe, as well as the right midbrain. These findings suggest a complex and bidirectional pattern of neurostructural changes in EC patients, reflecting both inflammatory-mediated compensatory increases and regional neurodegenerative decreases. The observed reductions in medial temporal lobe structures including the parahippocampal gyri and amygdala are particularly noteworthy given their well-established roles in memory encoding, emotional regulation, and contextual associative process (46–49). Atrophy in these regions has been consistently linked to cognitive impairment in other cancer-related and neurodegenerative conditions. Similarly, volume loss in the cerebellum posterior lobe, which contributes to cognitive coordination and fine motor control, and the midbrain, involved in dopaminergic signaling and sensorimotor integration, may underlie specific functional deficits reported in EC populations, such as gait instability, affective dysregulation, and executive dysfunction (50, 51). The concomitant presence of both increased and decreased GM volumes supports a model of cancer-related neural remodeling wherein neuroinflammatory processes and metabolic disturbances drive both maladaptive degeneration and compensatory plasticity, ultimately shaping the clinical presentation of CRCI in EC.

Exploratory partial correlation analysis in this study demonstrated a significant negative correlation between PLR and GM volume of the right basal ganglia in EC group. This significant negative correlation indicates that changed systemic inflammatory burden (as indexed by PLR) might be linked to structural neurodegeneration characterized by increased GM volume in the right basal ganglia nuclei, potentially mediated through neuroinflammatory cascades and microvascular dysfunction. The basal ganglia are a complex of subcortical nuclei critical for motor control and cognitive processing (32, 33). PLR is a widely utilized hematological biomarker of systemic inflammation (52). These findings collectively imply a potential neuroinflammatory mechanism wherein chronic inflammatory states (e.g., PLR) may drive neurodegenerative changes (e.g., GM) in functionally significant brain regions. Additionally, longitudinal studies are required to establish temporal causality between PLR fluctuations and GM volumetric dynamics in the future.

This study has several limitations that should be acknowledged. First, the relatively modest sample size may have limited the statistical power of our analysis. In future studies, we plan to expand the cohort and replicate these findings in larger, independent samples to enhance both statistical power and external validity. Secondly, this study did not include formal neurocognitive assessments. While the brain regions showing structural changes are implicated in functions known to be affected in CRCI, future studies that integrate comprehensive cognitive testing with neuroimaging and inflammatory biomarkers are essential to directly link these physiological changes to functional cognitive outcomes. Furthermore, while the primary analysis of this study is cross-sectional, a preliminary three-month longitudinal follow-up of a patient subset was conducted. The results from this analysis (provided in the Supplementary Materials) offer initial evidence of progressive GM changes, lending further support to the dynamic remodeling processes described. Nevertheless, longer-term follow-up studies remain essential to fully elucidate the trajectory and clinical implications of these neural adaptations. Another limitation of this study is the significant difference in gender distribution between the EC and HC groups, with fewer females in the EC group. Although age, gender, education, lifestyle factors (smoking and alcohol consumption), and BMI were included as covariates in the statistical models to minimize confounding effects, residual confounding cannot be fully excluded. Future studies should aim to recruit more balanced samples in terms of gender. Lastly, despite rigorous preprocessing and quality control, methodological factors cannot be fully excluded, so compensatory remodeling should be regarded as a plausible but not definitive explanation. Longitudinal and multimodal studies are required to validate these findings.

Our study systematically reveals the unique pattern and pathophysiological mechanism of central nervous remodeling in EC patients for the first time by integrating immune nutritional parameters and brain structural imaging data in multiple dimensions. We confirmed that the patients had significant immune imbalance (mononucleosis, lymphopenia and PLR/LMR abnormality) and nutritional metabolism disorder (PNI reduction), accompanied by extensive increase in GM volume in key brain regions, including bilateral basal ganglia, anterior and posterior central gyrus, temporal gyrus and occipital gyrus. This finding suggests that the essence of EC-related neurological changes may be an adaptive compensatory process driven by systemic inflammation (IL-6-mediated microglial activation) and metabolic disorders (albumin deficiency affecting neurotrophic factor transport). These results not only establish a theoretical model of the interaction of “ inflammation-metabolism-central remodeling”, but also provide a new target for clinical intervention - aiming to precisely maintain neurocognitive function by regulating the immune microenvironment (such as monocyte subsets), improving nutritional status (increasing PNI) and neuroprotective strategies (inhibiting glial cell overactivation). In future studies, multi-modal imaging combined with molecular marker tracking should be used to further analyze the characteristics of GM dynamic changes in specific brain regions of EC and their association with clinical outcome.

## Data Availability

The raw data supporting the conclusions of this article will be made available by the authors, without undue reservation.

## References

[1] Sung H Ferlay J Siegel RL Laversanne M Soerjomataram I Jemal A . Global cancer statistics 2020: GLOBOCAN estimates of incidence and mortality worldwide for 36 cancers in 185 countries. CA: Cancer J Clin. (2021) 71:209–49. doi: 10.3322/caac.21660, PMID: 33538338

[2] Chen W Zheng R Baade PD Zhang S Zeng H Bray F . Cancer statistics in China 2015. CA: Cancer J Clin. (2016) 66:115–32. doi: 10.3322/caac.21338, PMID: 26808342

[3] Bray F Ferlay J Soerjomataram I Siegel RL Torre LA Jemal A . Global cancer statistics 2018: GLOBOCAN estimates of incidence and mortality worldwide for 36 cancers in 185 countries. CA: Cancer J Clin. (2018) 68:394–424. doi: 10.3322/caac.21492, PMID: 30207593

[4] Abnet CC Arnold M Wei W-Q . Epidemiology of esophageal squamous cell carcinoma. Gastroenterology. (2018) 154:360–73. doi: 10.1053/j.gastro.2017.08.023, PMID: 28823862 PMC5836473

[5] Zhang L Zhou Y Cheng C Cui H Cheng L Kong P . Genomic analyses reveal mutational signatures and frequently altered genes in esophageal squamous cell carcinoma. Am J Hum Genet. (2020) 107:579. doi: 10.1016/j.ajhg.2020.08.012, PMID: 32888509 PMC7477001

[6] Von Ah D Habermann B Carpenter JS Schneider BL . Impact of perceived cognitive impairment in breast cancer survivors. Eur J Oncol Nurs. (2013) 17:236–41. doi: 10.1016/j.ejon.2012.06.002, PMID: 22901546

[7] Abdel-Latif MM Duggan S Reynolds JV Kelleher D . Inflammation and esophageal carcinogenesis. Curr Opin Pharmacol. (2009) 9:396–404. doi: 10.1016/j.coph.2009.06.010, PMID: 19596608

[8] Chela HK Gangu K Ertugrul H Juboori AA Daglilar E Tahan V . The 8th wonder of the cancer world: esophageal cancer and inflammation. Diseases. (2022) 10:44. doi: 10.3390/diseases10030044, PMID: 35892738 PMC9326664

[9] Calabrese V Santoro A Monti D Crupi R Di Paola R Latteri S . Aging and Parkinson's Disease: Inflammaging, neuroinflammation and biological remodeling as key factors in pathogenesis. Free Radical Biol Med. (2018) 115:80–91. doi: 10.1016/j.freeradbiomed.2017.10.379, PMID: 29080843

[10] Cervellati C Trentini A Pecorelli A Valacchi G . Inflammation in neurological disorders: the thin boundary between brain and periphery. Antioxidants Redox Signaling. (2020) 33:191–210. doi: 10.1089/ars.2020.8076, PMID: 32143546

[11] Kridin K Linder D Shalom G Piaserico S Babaev M Freud T . Psoriasis and dementia: A cross-sectional study of 121,801 patients. Acta Dermato-Venereologica. (2020) 100:adv00250. doi: 10.2340/00015555-3595, PMID: 32725254 PMC9207629

[12] An P Zhou X Du Y Zhao J Song A Liu H . Association of neutrophil-lymphocyte ratio with mild cognitive impairment in elderly Chinese adults: A case-control study. Curr Alzheimer Res. (2019) 16:1309–15. doi: 10.2174/1567205017666200103110521, PMID: 31902361

[13] Liu J-H Zhang Y-J Ma Q-H Sun H-P Xu Y Pan C-W . Elevated blood neutrophil to lymphocyte ratio in older adults with cognitive impairment. Arch Gerontology Geriatrics. (2020) 88:104041. doi: 10.1016/j.archger.2020.104041, PMID: 32155517

[14] Marsland AL Gianaros PJ Kuan DCH Sheu LK Krajina K Manuck SB . Brain morphology links systemic inflammation to cognitive function in midlife adults. Brain Behavior Immun. (2015) 48:195–204. doi: 10.1016/j.bbi.2015.03.015, PMID: 25882911 PMC4508197

[15] Chesnokova V Pechnick RN Wawrowsky K . Chronic peripheral inflammation, hippocampal neurogenesis, and behavior. Brain Behavior Immun. (2016) 58:1–8. doi: 10.1016/j.bbi.2016.01.017, PMID: 26802985 PMC4956598

[16] Dahnke R Yotter RA Gaser C . Cortical thickness and central surface estimation. NeuroImage. (2013) 65:336–48. doi: 10.1016/j.neuroimage.2012.09.050, PMID: 23041529

[17] Ashburner J . A fast diffeomorphic image registration algorithm. NeuroImage. (2007) 38:95–113. doi: 10.1016/j.neuroimage.2007.07.007, PMID: 17761438

[18] Han L-H Jia Y-B Song Q-X Wang J-B Wang N-N Cheng Y-F . Prognostic significance of preoperative lymphocyte-monocyte ratio in patients with resectable esophageal squamous cell carcinoma. Asian Pacific J Cancer Prevention: APJCP. (2015) 16:2245–50. doi: 10.7314/APJCP.2015.16.6.2245, PMID: 25824745

[19] Hirahara N Matsubara T Mizota Y Ishibashi S Tajima Y . Prognostic value of preoperative inflammatory response biomarkers in patients with esophageal cancer who undergo a curative thoracoscopic esophagectomy. BMC Surg. (2016) 16:66. doi: 10.1186/s12893-016-0179-5, PMID: 27650456 PMC5028997

[20] Hu G Liu G Ma J-Y Hu R-J . ). Lymphocyte-to-monocyte ratio in esophageal squamous cell carcinoma prognosis. Clinica Chimica Acta; Int J Clin Chem. (2018) 486:44–8. doi: 10.1016/j.cca.2018.07.029, PMID: 30028962

[21] Li K-J Xia X-F Su M Zhang H Chen W-H Zou C-L . Predictive value of lymphocyte-to-monocyte ratio (LMR) and neutrophil-to-lymphocyte ratio (NLR) in patients with oesophageal cancer undergoing concurrent chemoradiotherapy. BMC Cancer. (2019) 19:1004. doi: 10.1186/s12885-019-6157-4, PMID: 31655563 PMC6815405

[22] Singh R Mishra MK Aggarwal H . Inflammation, immunity, and cancer. Mediators Inflammation. (2017) 2017:6027305. doi: 10.1155/2017/6027305, PMID: 29234189 PMC5695028

[23] Zhou X-L Zhu W-G Zhu Z-J Wang W-W Deng X Tao W-J . Lymphopenia in esophageal squamous cell carcinoma: relationship to malnutrition, various disease parameters, and response to concurrent chemoradiotherapy. Oncologist. (2019) 24:e677–86. doi: 10.1634/theoncologist.2018-0723, PMID: 31040254 PMC6693723

[24] Qian B-Z Pollard JW . Macrophage diversity enhances tumor progression and metastasis. Cell. (2010) 141:39–51. doi: 10.1016/j.cell.2010.03.014, PMID: 20371344 PMC4994190

[25] Quail DF Joyce JA . Microenvironmental regulation of tumor progression and metastasis. Nat Med. (2013) 19:1423–37. doi: 10.1038/nm.3394, PMID: 24202395 PMC3954707

[26] Heneka MT Carson MJ El Khoury J Landreth GE Brosseron F Feinstein DL . Neuroinflammation in Alzheimer’s disease. Lancet Neurol. (2015) 14:388–405. doi: 10.1016/S1474-4422(15)70016-5, PMID: 25792098 PMC5909703

[27] Mantovani A Marchesi F Malesci A Laghi L Allavena P . Tumour-associated macrophages as treatment targets in oncology. Nat Rev Clin Oncol. (2017) 14:399–416. doi: 10.1038/nrclinonc.2016.217, PMID: 28117416 PMC5480600

[28] Capello M Vykoukal JV Katayama H Bantis LE Wang H Kundnani DL . Exosomes harbor B cell targets in pancreatic adenocarcinoma and exert decoy function against complement-mediated cytotoxicity. Nat Commun. (2019) 10:254. doi: 10.1038/s41467-018-08109-6, PMID: 30651550 PMC6335434

[29] Eskandari F Sternberg EM . Neural-immune interactions in health and disease. Ann New York Acad Sci. (2002) 966:20–7. doi: 10.1111/j.1749-6632.2002.tb04198.x, PMID: 12114255

[30] Arends J Bachmann P Baracos V Barthelemy N Bertz H Bozzetti F . ESPEN guidelines on nutrition in cancer patients. Clin Nutr (Edinburgh Scotland). (2017) 36:11–48. doi: 10.1016/j.clnu.2016.07.015, PMID: 27637832

[31] Jin S Cao S Xu S Wang C Meng Q Yu Y . Clinical impact of pretreatment prognostic nutritional index (PNI) in small cell lung cancer patients treated with platinum-based chemotherapy. Clin Respir J. (2018) 12:2433–40. doi: 10.1111/crj.12925, PMID: 30074685

[32] Dhawale AK Wolff SBE Ko R Ölveczky BP . The basal ganglia control the detailed kinematics of learned motor skills. Nat Neurosci. (2021) 24:1256–69. doi: 10.1038/s41593-021-00889-3, PMID: 34267392 PMC11152194

[33] Roth RH Ding JB . Cortico-basal ganglia plasticity in motor learning. Neuron. (2024) 112:2486–502. doi: 10.1016/j.neuron.2024.06.014, PMID: 39002543 PMC11309896

[34] Woodburn SC Bollinger JL Wohleb ES . The semantics of microglia activation: neuroinflammation, homeostasis, and stress. J Neuroinflamm. (2021) 18:258. doi: 10.1186/s12974-021-02309-6, PMID: 34742308 PMC8571840

[35] Mayer MG Fischer T . Microglia at the blood brain barrier in health and disease. Front Cell Neurosci. (2024) 18:1360195. doi: 10.3389/fncel.2024.1360195, PMID: 38550920 PMC10976855

[36] Gao C Jiang J Tan Y Chen S . Microglia in neurodegenerative diseases: mechanism and potential therapeutic targets. Signal Transduct Target Ther. (2023) 8:359. doi: 10.1038/s41392-023-01588-0, PMID: 37735487 PMC10514343

[37] Wang Y Fang J-L Cui B Liu J Song P Lang C . The functional and structural alterations of the striatum in chronic spontaneous urticaria. Sci Rep. (2018) 8:1725. doi: 10.1038/s41598-018-19962-2, PMID: 29379058 PMC5789061

[38] Germann J Chakravarty MM Collins DL Petrides M . Tight coupling between morphological features of the central sulcus and somatomotor body representations: A combined anatomical and functional MRI study. Cereb Cortex. (2020) 30:1843–54. doi: 10.1093/cercor/bhz208, PMID: 31711125 PMC7132904

[39] van Velzen MJM Pape M Sprangers MAG Van Kleef JJ Mostert B Beerepoot LV . Chemotherapy-induced peripheral neuropathy in patients with gastroesophageal cancer. J Natl Compr Canc Netw. (2024) 22:455–61. doi: 10.6004/jnccn.2024.7014, PMID: 38977016

[40] Cunningham G Zanchi D Emmert K Kopel R Van De Ville D Lädermann A . Neural correlates of clinical scores in patients with anterior shoulder apprehension. Med Sci Sports Exercise. (2015) 47:2612–20. doi: 10.1249/MSS.0000000000000726, PMID: 26110696

[41] Niddam DM Lee S-H Su Y-T Chan R-C . Altered cortical morphology in patients with chronic shoulder pain. Neurosci Lett. (2019) 712:134515. doi: 10.1016/j.neulet.2019.134515, PMID: 31560996

[42] Yue X Du Y . Altered intrinsic brain activity and regional cerebral blood flow in patients with chronic neck and shoulder pain. Polish J Radiol. (2020) 85:e155–62. doi: 10.5114/pjr.2020.94063, PMID: 32322322 PMC7172875

[43] Belin P Zatorre RJ Lafaille P Ahad P Pike B . Voice-selective areas in human auditory cortex. Nature. (2000) 403:309–12. doi: 10.1038/35002078, PMID: 10659849

[44] Wright TM Pelphrey KA Allison T McKeown MJ McCarthy G . Polysensory interactions along lateral temporal regions evoked by audiovisual speech. Cereb Cortex. (2003) 13:1034–43. doi: 10.1093/cercor/13.10.1034, PMID: 12967920

[45] Hebart MN Hesselmann G . What visual information is processed in the human dorsal stream? J Neurosci. (2012) 32:8107–9. doi: 10.1523/JNEUROSCI.1462-12.2012, PMID: 22699890 PMC6703654

[46] Kim J Pignatelli M Xu S Itohara S Tonegawa S . Antagonistic negative and positive neurons of the basolateral amygdala. Nat Neurosci. (2016) 19:1636–46. doi: 10.1038/nn.4414, PMID: 27749826 PMC5493320

[47] McGinnis GJ Friedman D Young KH Torres ER Thomas CR Jr. Gough MJ . Neuroinflammatory and cognitive consequences of combined radiation and immunotherapy in a novel preclinical model. Oncotarget. (2017) 8:9155–73. doi: 10.18632/oncotarget.13551, PMID: 27893434 PMC5354722

[48] Wang J Tambini A Lapate RC . The tie that binds: temporal coding and adaptive emotion. Trends Cognit Sci. (2022) 26:1103–18. doi: 10.1016/j.tics.2022.09.005, PMID: 36302710

[49] Fogwe LA Reddy V Mesfin FB . Neuroanatomy, hippocampus. In: StatPearls. StatPearls Publishing, Treasure Island (FL (2025)., PMID: 29489273

[50] Schmahmann JD Guell X Stoodley CJ Halko MA . The theory and neuroscience of cerebellar cognition. Annu Rev Neurosci. (2019) 42:337–64. doi: 10.1146/annurev-neuro-070918-050258, PMID: 30939101

[51] Chu HY Zhen X . Hyperpolarization-activated, cyclic nucleotide-gated (HCN) channels in the regulation of midbrain dopamine systems. Acta Pharmacol Sin. (2010) 31:1036–43. doi: 10.1038/aps.2010.105, PMID: 20676119 PMC4002296

[52] Zhong X Su T Yang Y Ye L Jiang L Qi Y . Platelet-lymphocyte and neutrophil-lymphocyte ratios are prognostic markers for pheochromocytomas and paragangliomas. J Clin Endocrinol Metab. (2023) 108:2230–9. doi: 10.1210/clinem/dgad149, PMID: 36917004

